# Generation of a recombinant chickenized monoclonal antibody against the neuraminidase of H9N2 avian influenza virus

**DOI:** 10.1186/s13568-020-01086-4

**Published:** 2020-08-20

**Authors:** Fei Wang, Yajuan Wang, Zhimin Wan, Hongxia Shao, Kun Qian, Jianqiang Ye, Aijian Qin

**Affiliations:** 1 Ministry of Education Key Lab for Avian Preventive Medicine, Yangzhou University, No.12 East Wenhui Road, Yangzhou, 225009 Jiangsu People’s Republic of China; 2 Jiangsu Co-innovation Center for Prevention and Control of Important Animal Infectious Diseases and Zoonoses, No.12 East Wenhui Road, Yangzhou, 225009 Jiangsu People’s Republic of China; 3 Joint International Research Laboratory of Agriculture and Agri-Product Safety of Ministry of Education of China, Yangzhou University, No.12 East Wenhui Road, Yangzhou, 225009 Jiangsu People’s Republic of China

**Keywords:** Recombinant chickenized monoclonal antibody, H9N2 AIV NA, NA inhibitory activity, Neutralizing ability

## Abstract

We previously reported a monoclonal antibody (mAb), 1G8, against the neuraminidase (NA) of H9N2 avian influenza virus (AIV) with significant NA inhibitory activity. To generate a recombinant chickenized mAb (RCmAb) against the NA of H9N2 AIV for passive immunization in poultry, the gene of the fragment of antigen binding (Fab) of mAb 1G8 was cloned and fused with the fragment crystallizable (Fc) gene of chicken IgY. The RCmAb 1G8 was expressed in COS-1 cells and could be detected in cell culture supernatant. The results of NA inhibitory activity tests of the RCmAb 1G8 in an enzyme-linked lectin assay (ELLA) and a microneutralization (MN) assay showed that the RCmAb 1G8 maintained significant NA inhibitory activity and neutralizing ability. This is the first chickenized antibody against AIV, which would be a good candidate for passive immunization in poultry.

## Key points

1. Sequences of variable region gene of mAb 1G8 against H9N2 NA were first identified.
2. The first RCmAb 1G8 against NA of H9N2 AIV was generated in this study.
3. RCmAb 1G8 maintains significant NA inhibitory activity and neutralizing ability.

## Introduction

H9N2 avian influenza virus (AIV) has been prevalent in China since the first outbreak in Guangdong Province in 1992. H9N2, a low-pathogenicity virus in domestic poultry, causes mainly respiratory symptoms, immunosuppression and a decline in egg production. Great economic losses can be caused when poultry are co-infected with other pathogenic organisms (Horwood et al. 2018; Pan et al. 2012). H9N2 is also a gene donor to other influenza viruses, such as H7N9, which is highly pathogenic to humans. Control of H9N2 in avian flocks is very important not only to animal health but also to public health.

AIV contains two surface proteins on the virus particle. One is haemagglutinin (HA), and the other is neuraminidase (NA). Both of them can induce neutralizing antibodies in chickens. Specific antibodies induced by NA contribute mainly to immunity by limiting viral replication and disease severity (Eichelberger et al. 2014). In addition, NA inhibitors, such as oseltamivir, zanamivir, peramivir, laninamivir, and a recently approved polymerase acidic (PA) inhibitor, baloxavir marboxil, are currently used to treat influenza virus infections. However, the emergence of antiviral drug resistance is a major concern for these inhibitors (Hussain et al. 2017).

Antibodies against NA can inhibit NA enzyme activity and neutralize viruses with antibody-dependent cell-mediated cytotoxicity (ADCC) and complement-dependent cytotoxicity (CDC) (Chen et al. 2018; Eichelberger and Wan 2014). Immunization with inactivated influenza vaccine induces much less NA-specific antibodies than natural infection (Chen et al. 2018). How to induce high concentrations of antibodies to NA in chickens needs to be investigated. Recently, breakthroughs in antibody phage display and monoclonal antibody (mAb) development have contributed to the development of humanized antiviral antibodies for passive immunization (Chen et al. 2017; Dong et al. 2013; Rudraraju and Subbarao 2018). However, few antibodies were chickenized for passive immunization of chickens (Roh et al. 2015). We previously reported a mouse-derived mAb 1G8, that has a significant inhibitory effect on the NA enzyme activity of H9N2 AIV (Wan et al. 2016). Additionally, mAb 1G8 could react with all H9N2 AIV strains isolated in eastern China. In the present study, we generated a recombinant chickenized monoclonal antibody (RCmAb) by fusing the gene of the fragment of antigen binding (Fab) of mAb 1G8 with the fragment crystallizable (Fc) gene of chicken IgY. This study is the first report of an RCmAb 1G8 against AIV, which would contribute to developing anti-AIV antibodies with heterogeneous antibodies for application in chicken passive immunization.

## Materials and methods

### Virus and cells

The H9N2 virus A/Chicken/Jiangsu/X1/2004 (X1) was isolated in Jiangsu Province, China. The virus was propagated in 9-day-old embryonated chicken eggs and preserved at − 70 °C. 1G8 hybridoma cells were screened from sp2/0 cells fused with spleen cells from BALB/C mice immunized with X1 virus, as previously reported (Wan et al. 2016). Madin-Darby canine kidney (MDCK) cells (ATCC, CCL-34) and COS-1 cells (ATCC, CRL-1650) were maintained in Dulbecco’s modified Eagle medium (DMEM) (Thermo Fisher Scientific, Massachusetts, USA) supplemented with 10% fetal bovine serum (FBS) (Thermo Fisher Scientific, Massachusetts, USA).

### RT-PCR and variable region sequence analysis

Total RNA of 1G8 hybridoma cells was extracted with an AxyPrep multisource total RNA miniprep kit (Corning, Jiangsu, China). Taking the cDNA from reverse transcription as template, the variable genes of the light chain or heavy chain were amplified with primers (Table 1). PCR products were purified with a gel extraction kit (QIAGEN, Dusseldorf, Germany) and then ligated with the pGEM-T easy vector (Promega, Wisconsin, USA) for sequencing.

**Table 1:** Primers used in amplifying variable region sequences of mAb 1G8

Primer name	Sequence^a^
VLF1	ATGGATTTTCAAGTGCAGATTTTCAG
VLF2	ATGKCCCCWRCTCAGYTYCTKGT
VLF3	ATGAAGTTGCCTGTTAGGCTGTTG
VLR	ACACTCATTCCTGTTGAAGCTCTTGAC
VHF1	CAGGTGCAGCTKMAGGAGTCA
VHF2	CAGGTCCARCTGCAGCAGYCT
VHF3	GARGTGAAGCTGGTGGARTCT
VHF4	GAGGTTCAGCTTCAGCAGTCT
VHR	ACAATCCCTGGGCACAATTTTC

^a^ In primers, K = G or T, W = A or T, R = A or G, Y = C or T, and M = A or C

The nucleotide sequences of the light chain (Accession number: MT490634) and heavy chain (Accession number: MT490635) were analyzed online by IgBLAST (NCBI). Then, the 3-D structure of the 1G8 Fab was constructed with ABodyBuilder (http://opig.stats.ox.ac.uk/webapps/newsabdab/sabpred/abodybuilder/, OPIG) by submitting the amino acid sequences of both the light chain and heavy chain.

### Plasmids construction

According to the types of top V genes in IgBLAST, we searched all V genes of the same type in the international immunogenetics information system (IMGT, www.imgt.org). To amplify Fab gene of mAb 1G8, primers were designed based on genes of Acession numbers AJ279029.1, BC049234.1, LC110289.1 in GenBank for light chain and Accession numbers BC085312.1, M19899.1, X05878.1 in GenBank for heavy chian (Table 2). Firstly, both light chain gene and Fd gene with signal peptide sequences were amplified from cDNA derived from hybridoma cells. And Fc gene of chicken IgY (Accession number: X07174.1) was amplified from cDNA derived from chicken spleen cells. Then the recombinant heavy chain gene was obtained by overlapping PCR with Fd gene and Fc gene of chicken IgY. Finally, the genes of light chain and the recombinant heavy chain were cloned into pCAGGS vector and named pCAGGS-LC and pCAGGS-FdchFc respectively.

**Table 2:** Primers used in plasmids construction

Primer name	Sequence
LCF-Sac	CGAGCTCATGAAGTCACAGACCCAGGTC
LCR-Nhe	CGGCTAGCCTAACACTCATTCCTGTTGAAGCTC
FdF-Sac	CGAGCTCATGAGAGTGCTGATTCTTTTGTGG
FdFcR	GGACCTGCACAATTTTCTTGTCCACCTTGGTGC
FdFcF	GGACAAGAAAATTGTGCAGGTCCTCCACCC
FcR-Nhe	CGGCTAGCTTATTTACCAGCCTGTTTCTGCAG

### Recombinant chickenized mAb 1G8 (RCmAb 1G8) expression

To generate the RCmAb, two plasmids, pCAGGS-LC and pCAGGS-FdchFc, were co-transfected into COS-1 cells at a ratio of 2:3 as reported (Pham et al. 2006; Smith et al. 2009). The culture medium of transfected cells was replaced by Opti-MEM (Thermo Fisher Scientific, Massachusetts, USA) after washing 3 times with phosphate-buffered saline (PBS) at 6 h post transfection. Plasmid pCAGGS was also transfected into COS-1 cells as negative control. The supernatants of transfected cells were respectively collected after 48 h post transfection for further assay.

### Western blot analysis

The supernatants of co-transfected cells were collected and treated with loading buffers with/without DL-dithiothreitol (DTT). Treated supernatants were used for SDS-PAGE and then transferred to nitrocellulose membranes (GE, Massachusetts, USA) for Western blot analysis as reported (Rüdiger Ridder 1995). Then, the membrane was directly incubated with an HRP-conjugated goat anti-chicken IgY (H+L) antibody (Jackson Immunoresearch, Pennsylvania, USA) for 1 h at 37 °C. After washing with PBST, the chemiluminescent signals were observed in a FluorChemE imaging system (Protein Simple, California, USA) covered with Clarity Max Western ECL Substrate (Bio-Rad, California, USA).

### Immunofluorescence assay (IFA)

To determine the reactivity of the recombinant antibody to H9N2 AIV, MDCK cells infected with X1 virus at 0.1 MOI were fixed by cold acetone-alcohol (3:2, vol/vol) at 72 h post infection. The plasmid pCAGGS-NA (X1) expressing NA of the X1 virus was transfected into COS-1 cells with Lipofectamine 2000 and fixed at 48 h post transfection. The RCmAb 1G8 was added to fixed plates and incubated for 30 min at 37 °C. After washing, goat anti-chicken IgY (H+L) conjugated with FITC (Jackson Immunoresearch, Pennsylvania, USA) was added and incubated for 30 min at 37 °C. The cells were finally washed with PBS and observed under fluorescence microscopy (Olympus, Tokyo, Japan).

### Enzyme linked lectin assay (ELLA)

The inhibition of the NA enzyme by the recombinant antibody was measured by ELLA (Couzens et al. 2014). First, fetuin was coated onto the surface of a 96-well plate. The supernatants of co-transfected cells were diluted from 2^− 1^ to 2^− 10^ and mixed with predetermined X1 virus. Purified mAb 1G8 was diluted and mixed with the virus in the same way, from an initial concentration of 20 µg/mL as a positive control. The mixtures were individually incubated in fetuin-coated wells for 16–18 h. After washing six times with PBST, horseradish peroxidase-conjugated peanut-agglutinin (PNA-HRP) (Sigma-Aldrich, Shanghai, China) was added and incubated at room temperature for 2 h. The plate was washed another six times with PBST, followed by the addition of tetramethylbenzidine (TMB) substrate and incubation for 15 min. The colour development was stopped with 1% SDS and detected by an ELISA reader (BioTek, Vermont, USA) at OD650.

### Microneutralization (MN) assay

The supernatant of co-transfected cells was diluted five fold with Opti-MEM containing 2 µg/mL trypsin treated with *N*-p-tosyl-l-phenylalanine chloromethyl ketone (TPCK) (Sigma-Aldrich, Shanghai, China). The supernatant of cells transfected with pCAGGS was diluted in the same way as the negative control. Purified mAb 1G8 at concentration of 5 µg/mL was used as a positive control. Then, all cells were separately incubated with 10 TCID_50_ of the X1 virus at 37 °C for 30 min and finally incubated with MDCK cells in 6-well plates. Three days later, the supernatants were collected and used for detection of the HA titer and TCID_50_. HA titer was measured with 0.5% chicken red blood cell as previously described (Jin et al. 2019). TCID_50_ of the virus in the supernatant was tested by previously described method (Jin et al. 2019) and calculated according to Reed-Muench assay.

## Results

### Characterization of variable region genes of mAb 1G8

**Fig. 1:**
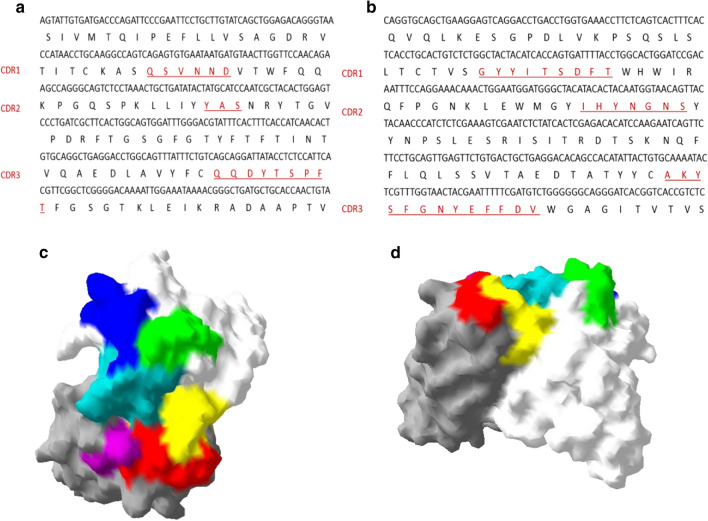
Sequences of the 1G8 variable region and 3-D structure model of the 1G8 Fab. The variable region sequences of the light chain (**a**) and heavy chain (**b**) are shown with underlined CDRs. The image of the Fab structure was constructed with DeepView software (Swiss PDB Viewer). The model is displayed in top view (**c**) and side view (**d**) with carton and surface rendering. The light chain is shown in white, with CDR1 marked in blue, CDR2 marked in cyan and CDR3 marked in green. The heavy chain is shown in grey, with CDR1 marked in red, CDR2 marked with purple and CDR3 marked with yellow

Sequences of the variable region of mAb 1G8 were analyzed online with IgBLAST (NCBI). The types of top V genes were IGKV6-32*01 for the light chain and IGHV3-1*01 for the heavy chain. Three complementarity-determining regions (CDRs) in the light chain and heavy chain were identified. For the light chain, CDR1 consists of “QSVNND”, and CDR2 consists of only three amino acids, “YAS” (Fig. 1a). The CDR3 of the light chain is made up of “QQDYTSPFT”. In the heavy chain, CDR1 consists of “GYYITSDFT”, and CDR2 consists of “IHYNGNS” (Fig. 1b). The longest CDR3 of the heavy chain is made up of “AKYSFGNYEFFDV”.

The 3-D structure of the 1G8 Fab was generated with ABodyBuilder (OPIG) by submitting amino acid sequences of the Fab of both the light chain and heavy chain. The shortest CDR2 of the light chain is located in the middle of the variable region and is tightly connected with the other CDRs (Fig. 1c, d). Interestingly, the longest CDR3 of the heavy chain is also located in the middle of the variable region, which is complementary to the shortest CDR2.

### RCmAb 1G8 was expressed in COS-1 cells

**Fig. 2:**
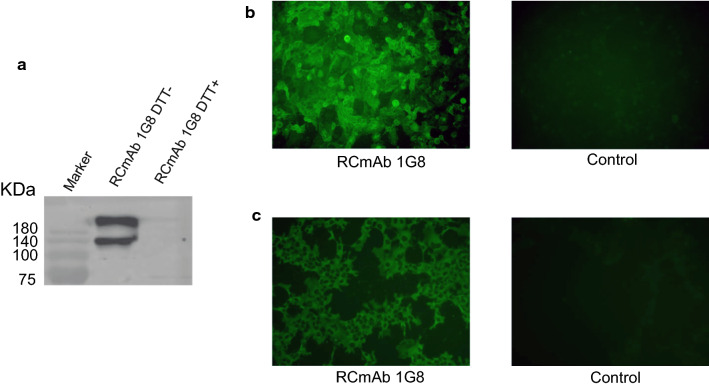
Production of RCmAb 1G8 against the NA of X1 virus. **a** Western blot analysis of the RCmAb 1G8 in the supernatant. Non-reducing sample (DTT−) and reducing sample (DTT+) of RCmAb 1G8 were tested. **b** IFA result of the RCmAb 1G8 reacting with X1 virus-infected MDCK cells. The supernatant of COS-1 cells transfected with pCAGGS was used as primary antibody in negative control. **c** IFA result of the RCmAb 1G8 reacting with pCAGGS-NA (X1) plasmid-transfected COS-1 cells. The supernatant of COS-1 cells transfected with pCAGGS was used as primary antibody in negative control

RCmAb 1G8 secreted into supernatant was analysed by Western blot. RCmAb 1G8 tetramers (over 180 kDa) and recombinant heavy chain dimers (130 kDa) were detected in the samples without DTT (Fig. 2a). This result indicated that the light chain and recombinant heavy chain could be expressed in the cells. The expressed protein could form tetramers and dimers and be excreted out of the cell. A weak band for the recombinant heavy chain monomer (65 kDa) was also detected in the samples with DTT (DTT+ in Fig. 2a).

### RCmAb 1G8 reacted with NA of H9N2

To determine whether the RCmAb 1G8 could specifically bind NA of H9N2 AIV, MDCK cells infected with X1 virus and COS-1 cells transfected with the pCAGGS-NA (X1) plasmid were incubated with the expressed RCmAb 1G8. Fluorescence imaging showed that the RCmAb 1G8 could react with the X1 virus (Fig. 2b) and NA protein of X1 virus (Fig. 2c), while the supernatant of COS-1 cells transfected with the pCAGGS plasmid showed no signal. Interestingly, the RCmAb 1G8 could also react with all H9N2 AIV strains isolated from 1999 to 2019 in eastern China, as mAb 1G8 did.

**Fig. 3:**
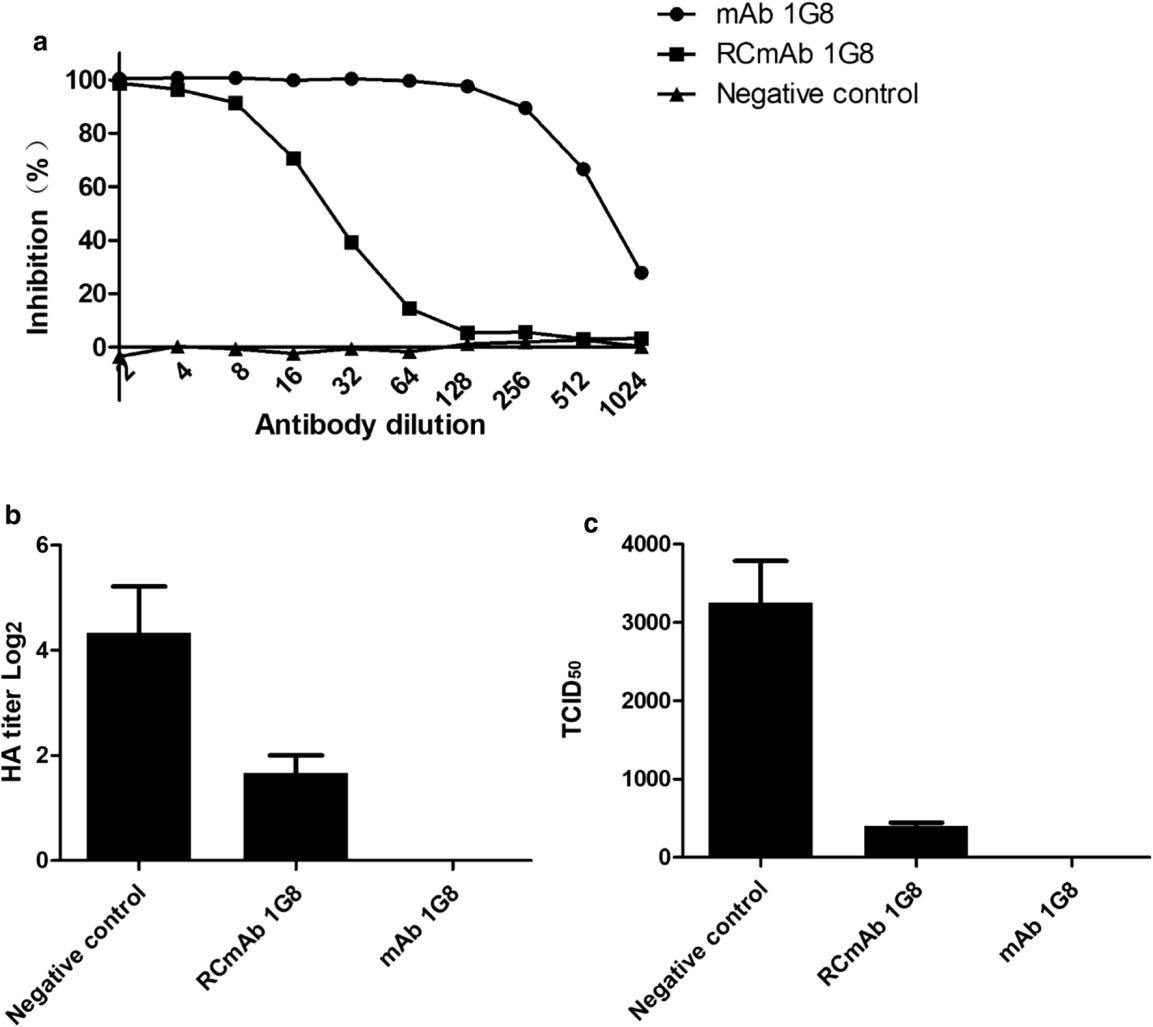
Inhibitory effect and neutralizing ability of the RCmAb 1G8 against NA. **a** Inhibition of NA activity measured via ELLA. RCmAb 1G8 was diluted from 2^− 1^ to 2^− 10^and incubated with X1 virus. Purified mAb 1G8 at initial concentration of 20 µg/mL was used as positive control. The supernatant of COS-1 cells transfected with pCAGGS was used as negative control. **b** HA titer and **c** TCID_50_ of X1 virus in MN assay incubated with RCmAb 1G8. mAb 1G8 at concentration of 5 µg/mL was used as positive control. The supernatant of COS-1 cells transfected with pCAGGS was used as negative control

NA activity inhibited by the RCmAb 1G8 was measured by ELLA. The results showed that similar to mAb 1G8, the RCmAb 1G8 could inhibit NA activity (Fig. 3a). The inhibition of NA activity by the RCmAb 1G8 in supernatant from co-transfected cells was equivalent to that by 0.156 µg/mL mAb 1G8 in the ELLA.

### RCmAb 1G8 neutralized H9N2 virus in MDCK cells

Strong neutralization of H9N2 AIV by the RCmAb 1G8 was also demonstrated in an MN assay. The HA titer of virus in the RCmAb 1G8 group (which is equivalent to 0.031 µg/mL mAb 1G8 in the ELLA) was only half of that in the negative control group (Fig. 3b). The mAb 1G8 group (5 µg/mL) was HA negative. Consistent with the HA results, no virus was detected in the supernatant medium of the mAb 1G8 group in the TCID_50_ test (Fig. 3c). The RCmAb 1G8 could also significantly inhibit the growth of the X1 virus, which indicated that NA activity was inhibited by the RCmAb 1G8 and that most nascent viruses could not be released from infected cells.

## Discussion

There are two surface glycoproteins on influenza A, HA and NA, which have essential functions in the influenza life cycle. HA mediates binding of the virus to the host cell, and NA cleaves off terminal sialic acids from glycans on the host cell and on the emerging virions, thereby enabling release of progeny viruses from the host cell. NA exhibits a slower antigenic drift that is generally discordant with that of HA (Kilbourne 1990). Therefore, antibody responses against NA typically show broader cross-reactivity than those against HA (Chen et al. 2018). NA is a validated drug target, and several small molecules that inhibit its activity are licensed as influenza therapeutics.

H9N2 AIV is a major epidemiological pathogen in domestic poultry in China and a great threat to domestic poultry and public health (Huang et al. 2013; Jiao and Liu 2016; Wang et al. 2015). Commercial H9N2 vaccines have been widely applied to elicit protective antibodies but have poor effects on preventing infection with current H9N2 AIV strains. Many broad-spectrum antibodies against AIV have been generated and applied in passive immunization in animal experiments as candidates for human-use antibody drugs (Doyle et al. 2013; Kallewaard et al. 2016). In this study, we generated the first chickenized mAb against the NA of H9N2 AIV.

Chickenized antibodies can be generated by fusion with the Fc of chicken IgY and CDR grafting. Although chickenized antibodies with CDR grafting were proven to have reduced immunogenicity in chickens, it is difficult to generate similar variable region structures because of conserved framework regions (FRs) in chicken IgY (Lee et al. 2017; Roh et al. 2015). The Fab gene of mouse-derived mAb 1G8 was fused with the chicken Fc gene and expressed in a secretory form in COS-1 cells. Antigen protein fusion with the Fc of chicken IgY has been previously reported to enhance antigen immunogenicity (Dong et al. 2016; Qin et al. 2016). The RCmAb 1G8 generated in this study can specifically react with the NA of H9N2 AIV, which may also have potential for NA antigen presentation in chickens. The RCmAb 1G8 produced in mammalian cells in this study was not sufficient for passive immunization in chickens, but it showed a great inhibitory effect on viruses in vitro, which indicates its potential application in the future.

## Data Availability

Not applicable.
